# Supplementary material for: Model of neural induction in the ascidian embryo
Source: PLoS Comput Biol. 2023 Feb 3;19(2):e1010335. doi: 10.1371/journal.pcbi.1010335 (PMC9931142; doi:10.1371/journal.pcbi.1010335)
Supplement: S1 Fig — A) ERK activities in the a6.5, a6.6, a6.7 and a6.8 cell types computed with the model, shown as Erk* values (left) or as Erkf values (right), with the relation between the two given by Eq (14) with A = 1850 and B = 155.11. Results are identical to those shown in Fig 2A where only the values of Erkf are indicated. (B) Nuclear dpERK IF signals in individual a-line cells of NVP-treated embryos are shown as a function of the relative area of cell surface contact with A-line cells in experiments and in the model. Experimental data are shown in grey (a6.8 cell type), green (a6.6 cell type), blue (a6.7 cell type), magenta (a6.5 cell type), predictions of the model in black. To consider the presence of NVP, [ephrin] = 0.001 in the model. Hill coefficient obtained by fitting the model prediction with a Hill function: 1.96. A = 2500 and B = 256.3 in Eq (14). The area shaded in grey represents the uncertainty on the model prediction. (C) Nuclear dpERK signals in the a6.5, a6.6, a6.7 and a6.8 cell types in NVP-treated embryos as measured in IF experiments (left) and computed with the model (right, Erkf values). Each point represents a single cell and modeling results are computed using the measured values of S1. To consider the presence of NVP, [ephrin] = 0.001 in the model. Means and standard deviations are shown in black. A = 2280 and B = 196.5 in Eq (14). (D) Injected/control ratios of nuclear dpERK signal in RGΔGAP injected half embryos. Left and right columns show ratios of activities of experimental dpERK and computed Erkf, respectively. Injection of p120RasΔGAP was modeled by considering Vrg = 0.01. A = 1850 and B = 155.11 in Eq (14). (PDF) [file pcbi.1010335.s001.pdf]

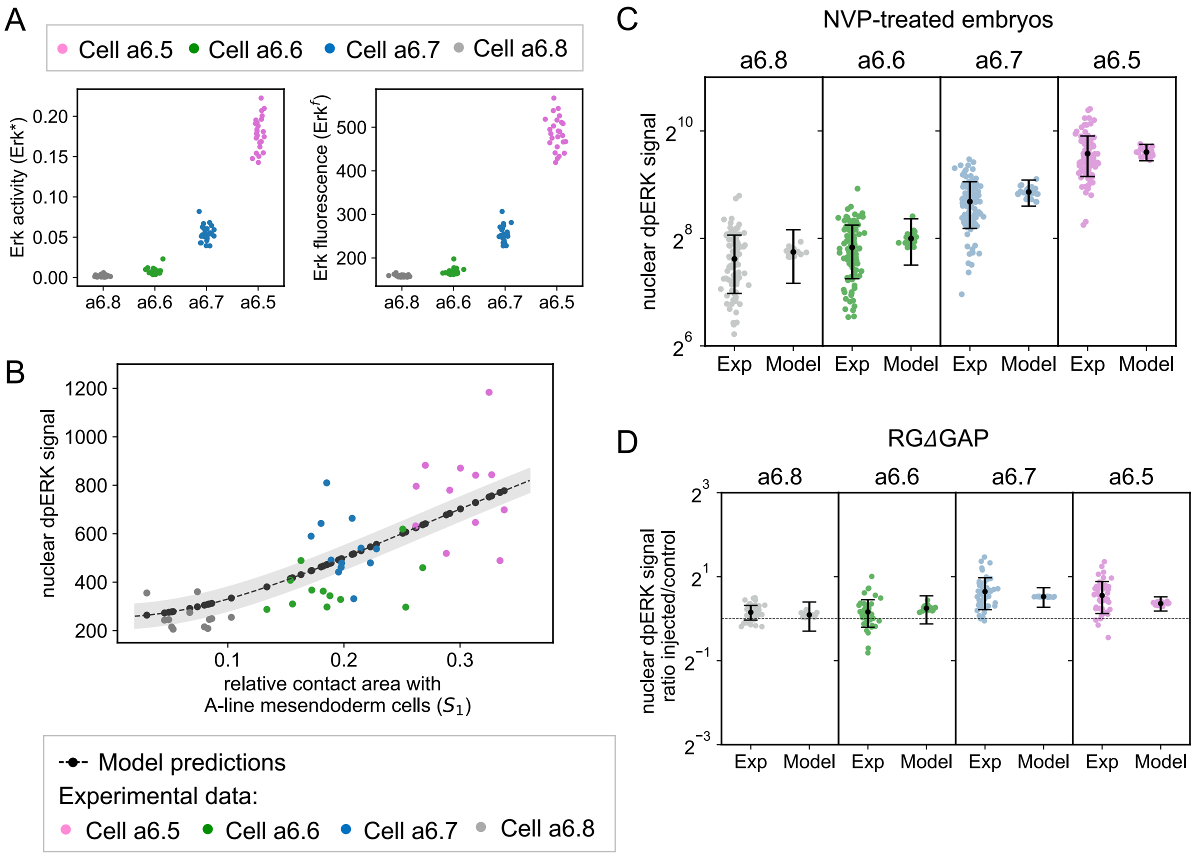

S1 Fig. Control of ERK activity by cell contact surfaces. A) ERK activities in the a6.5, a6.6, a6.7 and a6.8 cell types computed with the model, shown as Erk* values (left) or as Erk^f values (right), with the relation between the two given by Eq (14) with A=1850 and B= 155.11. Results are identical to those shown in Fig 2A where only the values of Erk^f are indicated**. (B)** Nuclear dpERK IF signals in individual a-line cells of NVP-treated embryos are shown as a function of the relative area of cell surface contact with A-line cells in experiments and in the model. Experimental data are shown in grey (a6.8 cell type), green (a6.6 cell type), blue (a6.7 cell type), magenta (a6.5 cell type), predictions of the model in black. To consider the presence of NVP, [ephrin]=0.001 in the model. Hill coefficient obtained by fitting the model prediction with a Hill function: 1.96. A=2500 and B=256.3 in Eq (14). The area shaded in grey represents the uncertainty on the model prediction. **(C)** Nuclear dpERK signals in the a6.5, a6.6, a6.7 and a6.8 cell types in NVP-treated embryos as measured in IF experiments (left) and computed with the model (right, Erk^f values). Each point represents a single cell and modeling results are computed using the measured values of S_1. To consider the presence of NVP, [ephrin]=0.001 in the model. Means and standard deviations are shown in black. A=2280 and B=196.5 in Eq (14). **(D)** Injected/control ratios of nuclear dpERK signal in RGDGAP injected half embryos. Left and right columns show ratios of activities of experimental dpERK and computed Erk^f, respectively. Injection of p120RasDGAP was modeled by considering V_{rg}=0.01. A= 1850 and B=155.11 in Eq (14).
