# Supplementary material for: Model of neural induction in the ascidian embryo
Source: PLoS Comput Biol. 2023 Feb 3;19(2):e1010335. doi: 10.1371/journal.pcbi.1010335 (PMC9931142; doi:10.1371/journal.pcbi.1010335)
Supplement: S2 Fig — (A) Left: drawing of 32-cell stage embryo, top-animal side view, bottom-vegetal side view. The ectoderm cells are encircled with a green dashed line (top) and the boundary between A- and B-line mesendoderm cells is marked with a green dotted line (bottom). The b-line ectoderm cells are colored following the color code indicated, with a-line ectoderm cells in white. The B-line mesendoderm cells are shaded with a greyscale heatmap depicting levels of FGF used in the model (A = B6.1 = 5; B6.2 = B6.4 = 0.7; B6.3 = 0.08). Middle graph shows the relative area of cell surface contact for each b-line cell with A-line mesendoderm and each B-line mesendoderm cell, with dots shaded with the grayscale FGF-heatmap. The right-hand graph shows the relative area of cell surface contact for each ectoderm cell with ephrin expressing ectoderm cells. In the graphs, each dot represents a single cell, n = 26 per cell type. (B) Nuclear dpERK signals in the b6.5, b6.6, b6.7 and b6.8 cell types as measured in IF experiments (left, n = 100 per cell type) and computed with the model (right, see ‘Model of Erk activity in b-line cells’ section). Each point represents a single cell and modeling results are computed using the measured values of S1 and S2 from panel (A). Means and standard deviations are shown in black. A = 1500 and B = 144.9 in Eq (14). (PDF) [file pcbi.1010335.s002.pdf]

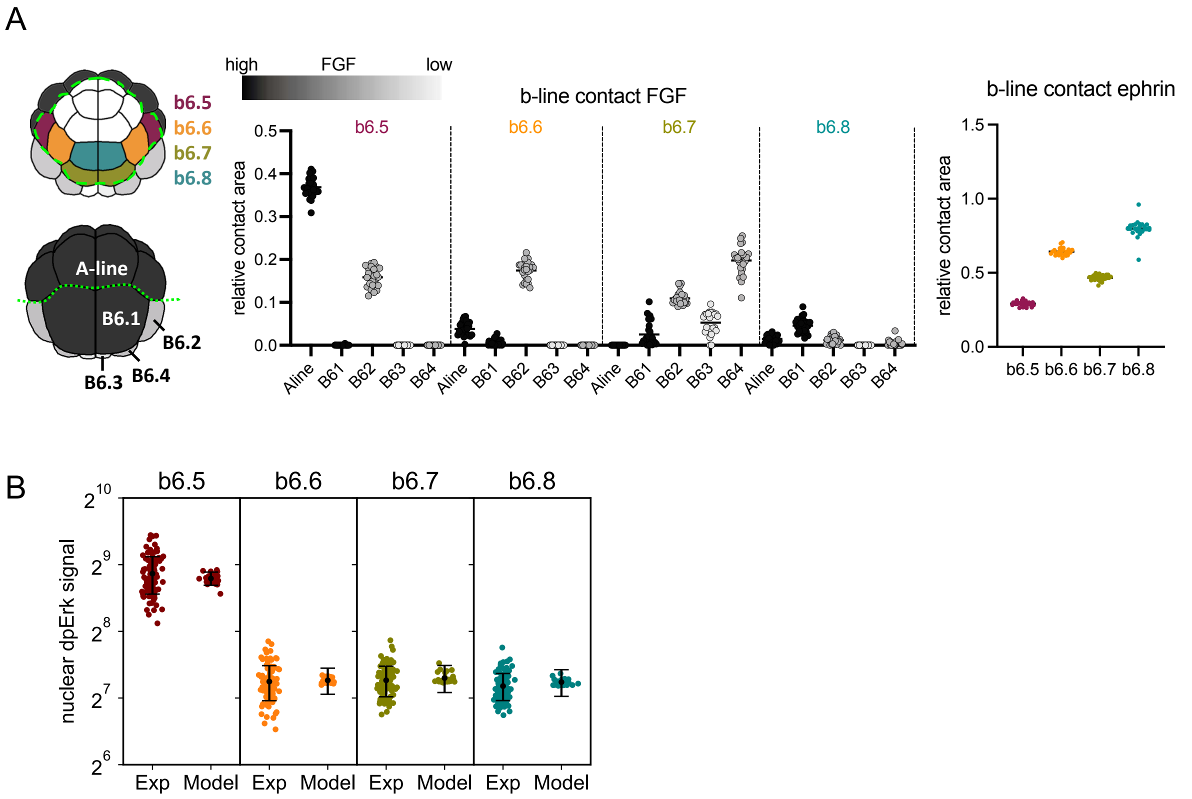

S2 Fig. Experimental data and predictions for b-line cells. (A) Left: drawing of 32-cell stage embryo, top-animal side view, bottom-vegetal side view. The ectoderm cells are encircled with a green dashed line (top) and the boundary between A- and B-line mesendoderm cells is marked with a green dotted line (bottom). The b-line ectoderm cells are colored following the color code indicated, with a-line ectoderm cells in white. The B-line mesendoderm cells are shaded with a greyscale heatmap depicting levels of FGF used in the model (A=B6.1= 5; B6.2=B6.4=0.7; B6.3=0.08). Middle graph shows the relative area of cell surface contact for each b-line cell with A-line mesendoderm and each B-line mesendoderm cell, with dots shaded with the grayscale FGF-heatmap. The right-hand graph shows the relative area of cell surface contact for each ectoderm cell with ephrin expressing ectoderm cells. In the graphs, each dot represents a single cell, n=26 per cell type. **(B)** Nuclear dpERK signals in the b6.5, b6.6, b6.7 and b6.8 cell types as measured in IF experiments (left, n=100 per cell type) and computed with the model (right, see *'Model of Erk activity in b-line cells'* section). Each point represents a single cell and modeling results are computed using the measured values of S_1 and S_2 from panel (A). Means and standard deviations are shown in black. A=1500 and B= 144.9 in Eq (14).
