# Supplementary material for: Model of neural induction in the ascidian embryo
Source: PLoS Comput Biol. 2023 Feb 3;19(2):e1010335. doi: 10.1371/journal.pcbi.1010335 (PMC9931142; doi:10.1371/journal.pcbi.1010335)
Supplement: S3 Fig — (A) Otx expression levels in the a6.5, a6.6, a6.7 and a6.8 cell types computed with the model, shown as O values (left) or as OtxsmFISH values (right), with the relation between the two given by Eq (18) with C = 66 and D = 2.75. Results are identical to those shown in Fig 3A where only the values of OtxsmFISH are indicated. (B) Relation between the concentrations of active activator of Otx expression (Ap, phosphorylated Ets1/2) and active repressor of Otx expression (I, unphosphorylated ERF2) and ERK activity (Erk*) in the model. The Hill coefficients of the curves were computed using relation (25). When combined with Eq (15) for Otx expression, the relation between O and Erk* takes the form of the curve shown in Fig 3F. (PDF) [file pcbi.1010335.s003.pdf]

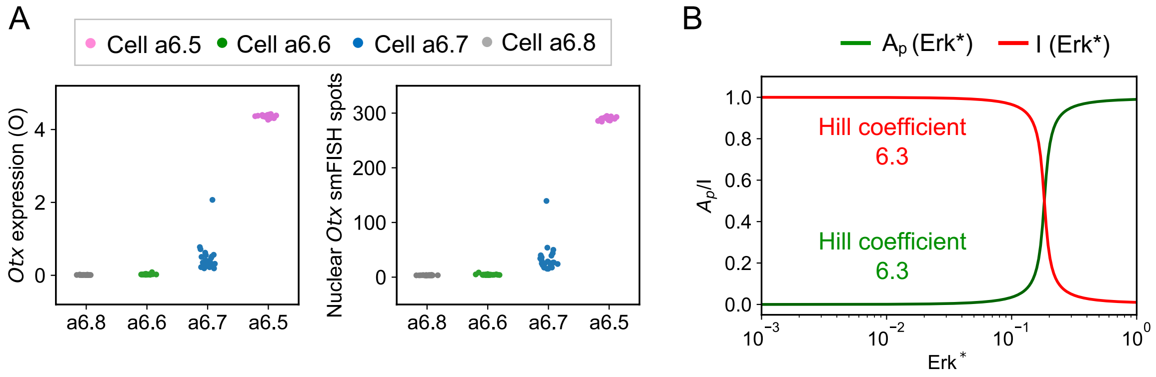

S3 Fig. **Dual control of *Otx* expression by antagonistic transcription factors. (A)** *Otx* expression levels in the a6.5, a6.6, a6.7 and a6.8 cell types computed with the model, shown as O values (left) or as Otx_{smFISH} values (right), with the relation between the two given by Eq (18) with C=66 and D=2.75. Results are identical to those shown in Fig 3A where only the values of Otx_{smFISH} are indicated. **(B)** Relation between the concentrations of active activator of *Otx* expression (A_p, phosphorylated Ets1/2) and active repressor of *Otx* expression (I, unphosphorylated ERF2) and ERK activity (Erk*) in the model. The Hill coefficients of the curves were computed using relation (25). When combined with Eq (15) for *Otx* expression, the relation between O and Erk* takes the form of the curve shown in Fig 3F.
