# Supplementary material for: Model of neural induction in the ascidian embryo
Source: PLoS Comput Biol. 2023 Feb 3;19(2):e1010335. doi: 10.1371/journal.pcbi.1010335 (PMC9931142; doi:10.1371/journal.pcbi.1010335)
Supplement: S4 Fig — (A) Otx expression in dnFGFR (upper left), Eph3ΔC (upper right) and RGΔGAP (lower left) injected embryo halves. In each plot, left and right columns show experimental Otx smFISH spots and computed OtxsmFISH, respectively. Injection of dnFGFR, Eph3ΔC and RGΔGAP were modeled by considering Rtot = 100, Qtot = 10, and Vrg = 0.01, respectively. In Eq (18), C = 92 for the control embryos and for dnFGFR injected embryos, C = 95 for Eph3ΔC injected embryos and C = 122 for RGΔGAP injected embryos. D = 1.5 for the control and dnFGFR injected embryos, D = 2.71 for Eph3ΔC and D = 1.61 for RGΔGAP injected embryos. (B) Effect of the ephrin/Eph inhibitor NVP on Otx expression in the four cell types. On the left, NVP-treated embryos; on the right, embryos treated with NVP and with the MEK inhibitor U0126 0.2 μM. In both cases, experimental Otx smFISH spots are shown on the left and computed OtxsmFISH values on the right. Each dot represents a single cell. NVP and moderate U0126 treatment are simulated by considering [ephrin] = 0.001 and Kerk = 0.6, respectively. In Eq (18), C = 94, D = 1.2. The data shown are the same as the ones presented in Fig 3B and 3C. (PDF) [file pcbi.1010335.s004.pdf]

A

B

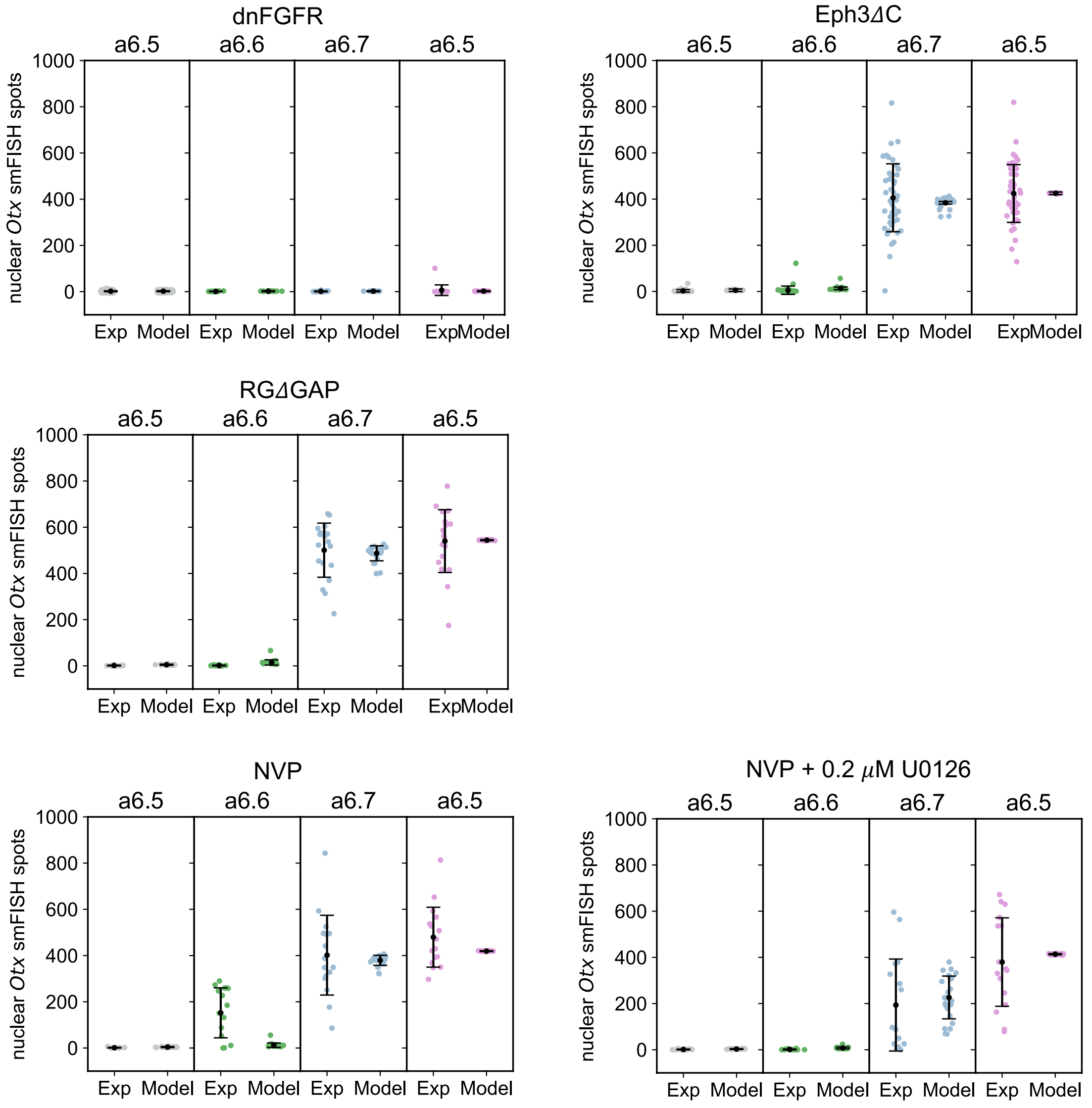

S4 Fig. Extended representation of the data shown in Fig 3B and 3C. (A) *Otx* expression in dnFGFR (upper left), Eph3ΔC (upper right) and RGΔGAP (lower left) injected embryo halves. In each plot, Left and right columns show experimental *Otx* smFISH spots and computed Otx_{smFISH}, respectively. Injection of dnFGFR, Eph3DC and RGΔGAP were modeled by considering R_{tot}=100, Q_{tot}=10, and V_{rg}=0.01, respectively. In Eq (18), C=92 for the control embryos and for dnFGFR injected embryos, C=95 for Eph3ΔC injected embryos and C=122 for RGΔGAP injected embryos. D=1.5 for the control and dnFGFR injected embryos, D=2.71 for Eph3ΔC and D=1.61 for RGΔGAP injected embryos. **(B)** Effect of the ephrin/Eph inhibitor NVP on *Otx* expression in the four cell types. On the left, NVP-treated embryos; on the right, embryos treated with NVP and with the ERK inhibitor U0126 0.2 μM. In both cases, experimental *Otx* smFISH spots are shown on the left and computed Otx_{smFISH} values on the right. Each dot represents a single cell. NVP and moderate U0126 treatment are simulated by considering [ephrin]= 0.001 and K_{erk} = 0.6, respectively. In Eq (18), C = 94, D=1.2. The data shown are the same as the ones presented in Fig 3B and 3C.
