# Supplementary material for: Model of neural induction in the ascidian embryo
Source: PLoS Comput Biol. 2023 Feb 3;19(2):e1010335. doi: 10.1371/journal.pcbi.1010335 (PMC9931142; doi:10.1371/journal.pcbi.1010335)
Supplement: S5 Fig — Because the boundaries that can be reached by T when varying FGF or ephrin are different, ERK activity does not vary between the same limits. Thus, the relation between Erk* and the [FGF] or [ephrin] is different in the two situations, as shown in Fig 6C. Values of the parameters used: Vs = Vrg = 1, K1 = K2 = 0.5, Krg = Ks = 1200, [FGF] or [ephrin] = 5, Kd = Ke = 25, RT = QT = 2000, S1 = S2 = 0.5, Kb = 10−6. (PDF) [file pcbi.1010335.s005.pdf]

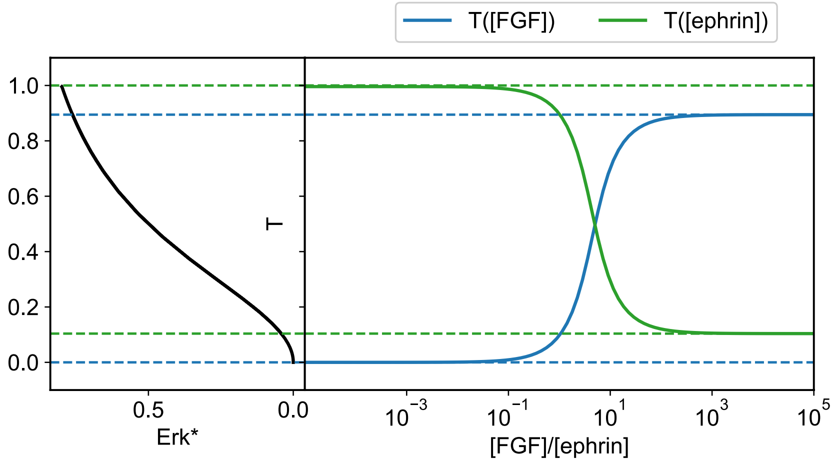

S5 Fig. Asymmetric dependence of the fraction of Ras-GTP (T) on the SOS (FGF) and RasGAP (ephrin) pathways. Because the boundaries that can be reached by T when varying FGF or ephrin are different, ERK activity does not vary between the same limits. Thus, the relation between Erk* and the [FGF] or [ephrin] is different in the two situations, as shown in Fig 6C. Values of the parameters used: V_s= V_{rg}=1, K_1= K_2=0.5, K_{rg}= K_s=1200, [FGF] or [ephrin]=5, K_d= K_e=25, R_T= Q_T=2000, S_1= S_2= 0.5, K_b=10^{-6}.
