# Supplementary material for: Model of neural induction in the ascidian embryo
Source: PLoS Comput Biol. 2023 Feb 3;19(2):e1010335. doi: 10.1371/journal.pcbi.1010335 (PMC9931142; doi:10.1371/journal.pcbi.1010335)
Supplement: S6 Fig — Right: Heatmaps showing the Hill coefficients of the relation between Otx and Erk* when changing the values of the KMMi in Eqs (16–17). (PDF) [file pcbi.1010335.s006.pdf]

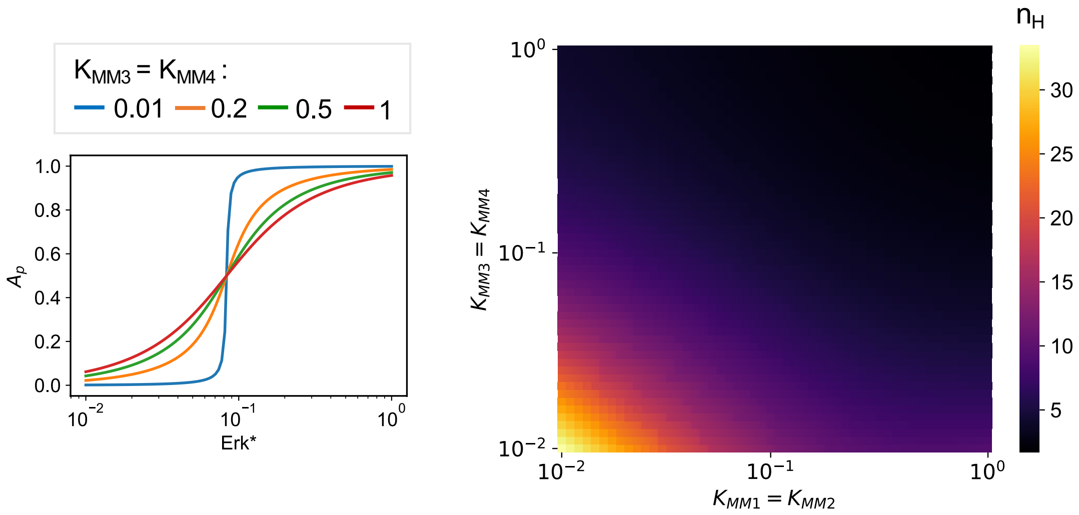

S6 Fig. Left: Effect of changing the K_{MMi} in Eq (16) on the relation between the fraction of phosphorylated activator, A_p and active ERK, Erk*. Right: Heatmaps showing the Hill coefficients of the relation between *Otx* and Erk* when changing the values of the K_{MMi} in Eqs (16-17).
