# Supplementary figures and images for: Model of neural induction in the ascidian embryo
Source: PLoS Comput Biol. 2023 Feb 3;19(2):e1010335. doi: 10.1371/journal.pcbi.1010335 (PMC9931142; doi:10.1371/journal.pcbi.1010335)

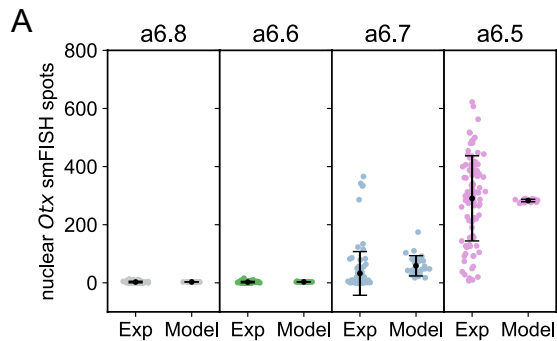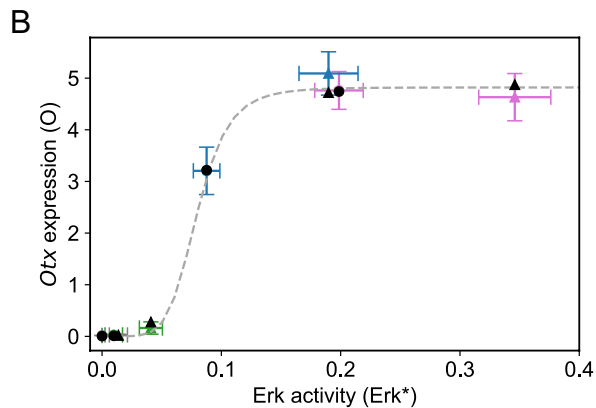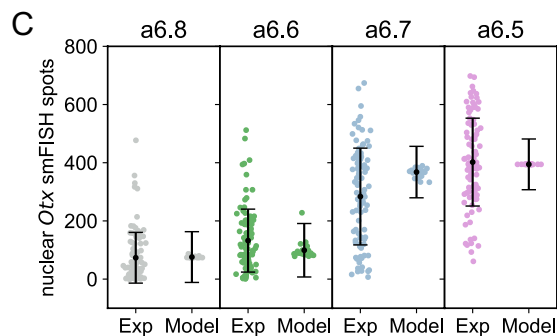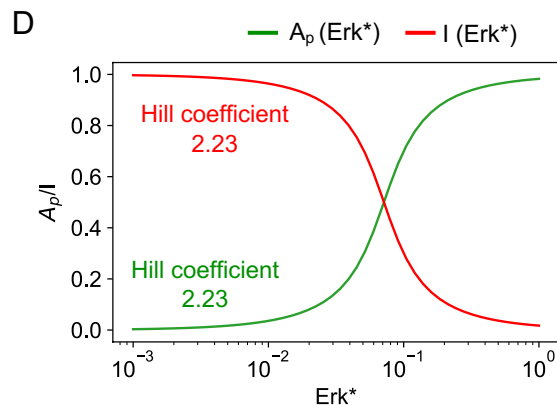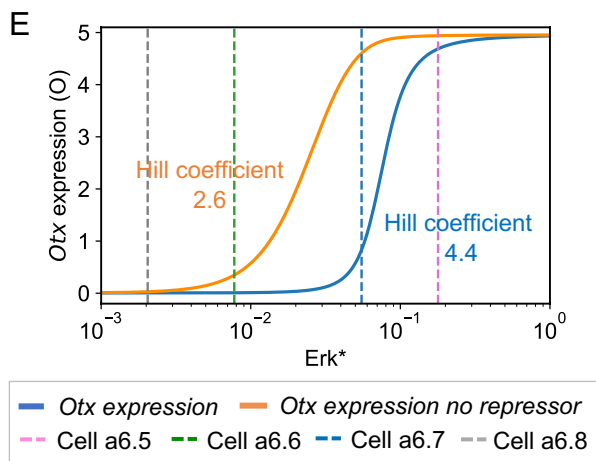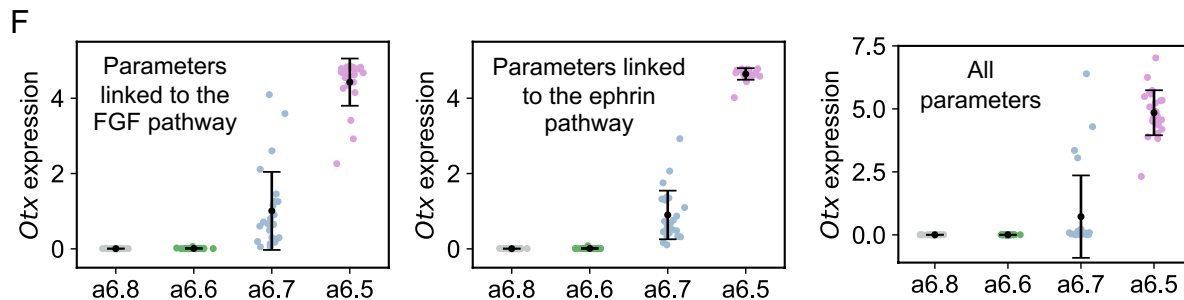

Supplement: S7 Fig — Otx expression levels were computed considering cooperativity in Otx activation (see section ‘Cooperativity in Otx activation’) Standard parameter values in Table 1 or: KMM1 = KMM2 = KMM3 = KMM4 = 0.3, kMM3 = kMM1 = 14. (A) Levels of Otx expression in the a6.5, a6.6, a6.7 and a6.8 cell types as measured by single molecule fluorescence in situ hybridization (smFISH, left) and computed with the model (right, OtxsmFISH). Each point represents a single cell and modeling results are computed using the measured values of S1. Means and standard deviations are shown in black. C = 60 and D = 2.75 in Eq (18). (B) Otx expression as a function of ERK activity (Erk*) in the four cell types (colored points and triangles) and computed with the model (OtxsmFISH, black points and triangles) considering cooperativity in Otx activation (see section ‘Cooperativity in Otx activation’). In the two cases, dots indicate the control while triangles indicate the ephrin-inhibited embryos. The experimental data were fitted by a Hill function, best-fit Hill coefficient = 6.09. For the experimental points, the value of Erk* corresponding to each cell was obtained by inversion of Eq (14), with A = 3200 and B = 0. The experimental value of O corresponding to each cell was obtained by inversion of Eq (18), with C = 110 and D = 0. Modelled Otx outputs were computed using the experimentally measured Erk* estimates as inputs. (C) Levels of Otx expression in embryos from eggs injected with an ERF2-morpholino to inhibit translation of ERF2 (left) and modelled with I = 0.01 (Eq (15)) (right). C = 73. 4 and D = 65 in Eq (18). (D) Relation between the concentrations of active activator (Ap, phosphorylated Ets1/2) and active repressor (I, unphosphorylated ERF2) and ERK activity in the cooperativity model. The Hill coefficients of the curves were computed using relation (25). (E) Otx expression (O) as a function of Erk*, computed with the cooperativity model considering the presence (blue line, Hill coeffici [file pcbi.1010335.s007.pdf]
