# Supplementary material for: Model of neural induction in the ascidian embryo
Source: PLoS Comput Biol. 2023 Feb 3;19(2):e1010335. doi: 10.1371/journal.pcbi.1010335 (PMC9931142; doi:10.1371/journal.pcbi.1010335)
Supplement: S8 Fig — Heatmaps showing the Hill coefficients of the relationship between, Otx and Erk* when changing values for KMMi in Eqs (16–17) using the cooperativity model (Eq (21)). (PDF) [file pcbi.1010335.s008.pdf]

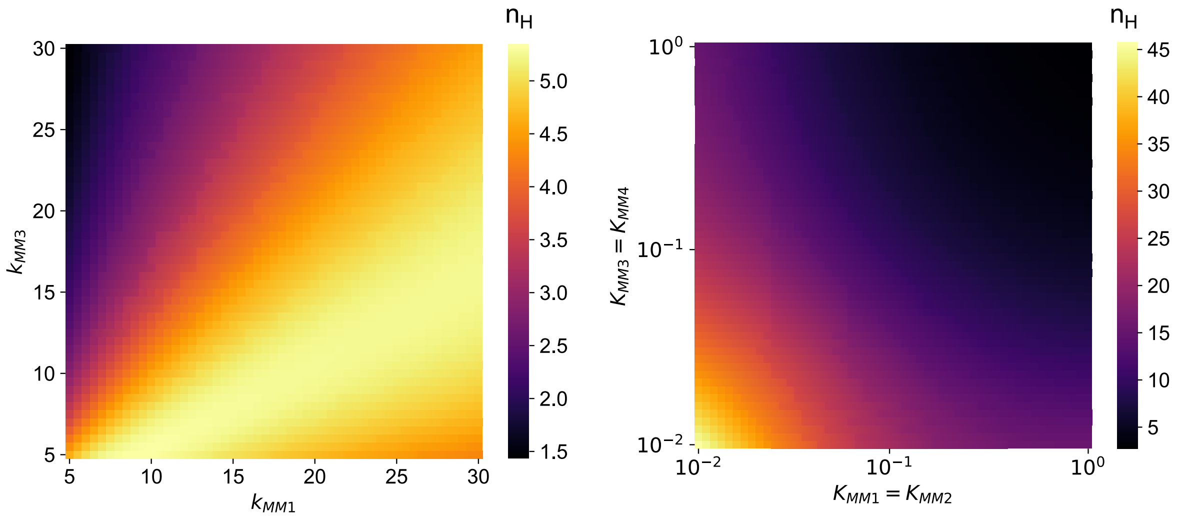

S8 Fig. Left: Heatmap showing the Hill coefficients of the relation between *Otx* and Erk* when changing the values of k_{MMi} in Eqs (16-17) followed by the cooperativity model (Eq (21)). Right: Heatmaps showing the Hill coefficients of the relationship between, *Otx* and Erk* when changing values for K_{MMi} in Eq (16-17) using the cooperativity model (Eq (21)).
