# Supplementary material for: Model of neural induction in the ascidian embryo
Source: PLoS Comput Biol. 2023 Feb 3;19(2):e1010335. doi: 10.1371/journal.pcbi.1010335 (PMC9931142; doi:10.1371/journal.pcbi.1010335)
Supplement: Attachment — Submitted filename: PCOMPBIOL-D-22-00981_ASCIDIAN_NEURAL_INDUCTION.pdf [file pcbi.1010335.s009.pdf]

The proposed computational study primarily uses the previously published mathematical models for the ERK pathway to demonstrate a contact-area-based neural induction in an ascidian embryo. Here, the authors also conducted a sensitivity analysis to establish the robustness of the contact area-based cell specification. Besides, the work also analyzes the role of the two competing pathways in the underlying gene regulation that generates an ON/OFF behavior of the neural induction in ascidian. Authors use the experimental outcome of their previous work and combine the previously developed independent mathematical modules to predict a set of new observations.

Authors claimed that the proposed mathematical model is an "enhanced, full mathematical description of FGF and ephrin-signaling control", but it appears difficult to differentiate the new modeling contribution made in the paper from their previously published work. Also, the model excludes the extracellular signaling dynamics of FGF and the subsequent intracellular signaling cascade. As a result, a complete top-down dynamic description of neural induction of neural precursor cells is absent in this mathematical model. Also, none of the previous assumptions are relaxed to conduct an exhaustive search of alternative hypotheses. The models used here are grossly simplified to be treated as a detailed mechanistic representation of the underlying processes. A set of specific observations about the manuscript is as follows:

Major Points:

One major weakness of the paper is the lack of novelty in the computational model, as it does not add a novel perspective compared to the mathematical model introduced in earlier works. For instance, extracellular signaling dynamics, both temporal and spatial, are discarded following similar assumptions made in their previously published manuscript. I think the extracellular signaling dynamics, at least in its simplified form, should be considered to differentiate the authors' contributions from previous works. Overall, the paper needs to capture more temporal and spatial (at least for FGF) variation through in-silico modeling, data generation, and analysis.

Downstream activation of signaling pathways and the robustness rely directly on how the extracellular ligand-receptor-regulator dynamics evolve temporarily and spatially. In the process, free-ligand availability is important and often varies spatially over a region because of the heterogeneous extracellular environment. For instance, surface heterogeneity may affect receptor level for the free ligand to bind, or the numerous other signaling regulators may hinder the availability of free ligand to the receptors. These all affect downstream signaling activation, and a

trace of it could be considered in the proposed computational study. For instance, a variation in FGF profile from its currently assumed uniform level requires further exploration to see the changes shape up as the downstream pathways are activated. The four cells of interest are exposed to uniform and constant FGF concentration. But it would be interesting to see the response of the graded FGF exposure to the cells of interest.

How about varying embryo volume or cell volume, or both, to see if the EGF>ERK>ETS>Otx expression remains invariant? Authors may consider using relative-volume to normalize any volumetric variation at the cellular or embryo level and demonstrate the relationship between the contact areas and the underlying neural induction.

[Line 243 to 244, Line 285 to 285] How a moderately nonlinear relationship between surface contacts with FGF-expressing cells and ERK activation transforms into a highly nonlinear relation between Otx expression and ERK activity may depend on numerous other factors that are not considered in the analysis. For instance, the intracellular dynamics are assumed in this study to be linearly cascading down to the next level, which is just an assumption considered previously and not explored furter in this mechanistic study. Authors could expand the analysis to accommodate the intracellular dynamics to channel down the nonlinearity part to the interplay between ERF, ETS, Ephrin, and GATA interactions. Interestingly, GATA is known to promote ERK signaling, thereby a potent candidate for becoming the source of additional nonlinearity.

[Line 178 to 185] The modeling assumptions and other descriptions require further clarification. For instance, exact simulation details to achieve steady-state solutions of Eq. 1-13 that include both Ordinary Differential Equations (ODE) as well as algebraic expressions for the downstream activation are missing. How long the dynamics were allowed to evolve to reach a steady state? Also, authors should clarify the units of each quantity being computed or measured. If the analysis considers dimensionless form, they should explicitly mention that (See line 186 to 187 of the manuscript, computed Erk* is dimensionless).

[Line 194 to 196] The model used an extracellular FGF level of KD = 25/5, which produced a maximum ERK activation level of 0.18 in an a6.5 cell. Any rationale for the specific KD/5? Is it to avoid receptor saturation? At what level do we encounter receptor saturation? Does it have anything to do with the areas of cell-contacts we have? Also, the dependency of FGF level and the maximal ERK activation could be explored in addition to an artificial overexpression of FGF

simulation. I think the authors could have exploited the flexibility of an in silico analysis to provide a more detailed picture of extracellular and intracellular coupling of FGF-mediated ERK activation and the subsequent activation of the downstream.

[Line 230 to Line 232] Authors report a reduction in ERK activity in cells for a reduction in FGF receptors. Is this reduction occurs because of receptor saturation? If yes, authors may want to see the dose-dependent response of the receptor occupancy, a mechanistic model of the FGF, FGFR, and the subsequent activation of the downstream would provide a better understanding of the underlying reasons.

[Line 323 to Line 324] Authors reported a stronger control by FGF-receptor pathway on the Otx activation than the control exerted by the ephrin/Eph. Can we have a ratiometric presentation of this stronger for parameters affecting the strength of the control? Is the ephrin/Eph is dispensible at any condition? For instance, overexpression of EGF or through the role of GATA that strengthens the ERK activation further? What happens to this stronger control if concentrations of EGF and/or ephrin changes in combination?

[Line 349 to Line 352] Sensitivity analysis on surface contact of ephrin-expressing cells and ephrin-concentration requires further clarification. How a difference between hill-coefficient manifests the higher sensitivity for surface contact is not clear from the manuscript. Perhaps, the authors consider the level of cooperativity exerted by each of these competing factors. If so, a better explanation would help readers to relate the hill-coefficient difference to the degree of sensitivity. Also, modeling details of the sensitivity analysis are insufficient for readers to follow and reproduce the study.

Authors could consider a rigorous kinetic screen (over a physiologically relevant parameter range) in the intracellular regulation of Otx expression through the two competing pathways– ERF2 and ETS. The kinetic dependence of the transformation of moderately nonlinear extracellular coupling of FGF and ERK to a strong nonlinear dependence of Otx on the ERK activation would be interesting to see.

[Line 520 to 522] Authors claimed to combine the previously developed three modules and calibrated with previously obtained experimental data. This does not portray any clear contribution

in the mechanistic model building, as the model remains incapacitate to portray the signaling dynamics.

[Line 527 to 528] Were the FGF and Eph receptor density similar? Did the authors consider alternative combinations of receptor density to capture the impact on the interplay between the FGF and ephrin signaling? It is important.

Table 1 data does not show units of any quantity. The authors claim that the data obtained through the best agreement with experimental observations, most (if not all) of which are from the previously published paper. The sensitive parameters could be considered in range to perform a kinetic screen (as suggested above) for an exhaustive in-silico response of the system. Precisely, the KD for phosphorylation of ETS turns out to be about 1200 (KD = K_on/K_off) as per the reported value in the paper, which indicates a very low binding affinity. A screen over a wide KD range may explain the role of Phosphorylation process (details of which are discarded in the mathematical model) better in the final ON/OFF induction. Also, receptor count requires cellular volume, which is not clarified anywhere in the manuscript.

A few additional short points:

- Cell volume used to count R_{T} = 2000 to be clarified.
- Knockdown of receptor level considers 100 receptors for FGF and 10 for Ephrin are different without any explanation. Why they are different? What were the normal receptor density considered for FGF and Ephrin? **[Line 272 to 276]**
- Referring to equation in the manuscript does not follow any uniform style– sometimes it is Eq (X), Eq. X. Authors should choose a uniform style.
- **In line 647**, a bracket is missing for the maximal Otx expression rate equation.
- Authors could arrange the mathematical equations considering their relevance (extracellular, intracellular) following their appearance in the top-down hierarchy of the singaling pathways.
- Figure 5 is difficult to follow– authors should clarify further the way the hill-coefficient difference for alternative parameters are summing up to higher degree of sensitivity.
