# Supplementary material for: Model of neural induction in the ascidian embryo
Source: PLoS Comput Biol. 2023 Feb 3;19(2):e1010335. doi: 10.1371/journal.pcbi.1010335 (PMC9931142; doi:10.1371/journal.pcbi.1010335)
Supplement: Attachment — Submitted filename: Response_Bettoni.pdf [file pcbi.1010335.s010.pdf]

Manuscript "Model of neural induction in the ascidian embryo" by Bettoni et al.
Response to the Referees

We thank all 3 reviewers for their careful reading and constructive suggestions. We have taken most of them into account in the new version of the manuscript, as detailed here below. Modified text is indicated in red in the new version of the manuscript.

Reviewer #1

Minor concerns:

1. The main criticism that the authors are surely going to get is direct comparisons with the Willaume et al 2021 Dev Cell paper. This can't be avoided since that is the dataset they are using. Nevertheless, I would recommend removing figure 1B since it is a complete copy and paste from the previous paper and replacing it with a new schematic.
The experimental data and the different modules that constitute the current model have indeed already been presented in Williaume et al., 2021 as was stated in the last paragraph of the Introduction of the manuscript. We have now further clarified the fact that most experimental observations used in the current study derive from Williaume et al., 2021 (see lines 128-130). The modelling modules that were presented in this previous publication were not integrated together, nor calibrated on experimental data, and were used as tools to test concepts and propose hypotheses directly based on experimental observations. As is often the case, it was not possible to present a full analysis of the complete computational model in the first, mostly experimental, publication. As suggested, we have also replaced old Fig. 1B by a scheme, which is also from Williaume et al, 2021. We agree that this figure is based on Williaume et al, 2021 but we feel it contains essential information required to understand the rest of the manuscript.

2. Otx is expressed in the b6.5 cells (as well as the B6.1/2/4 cells, but these are surely regulated by other processes) at the 32 cell stage. Surely the author's model can also account for the expression in the b6.5 cells? This would do a lot to show the usefulness of the model beyond the Willaume et al 2021.
We thank the Referee for this nice suggestion. To this end, we have used a new set of experimental measurements of ERK activation levels in the b-line ectoderm cells and of areas of their cell contact surfaces with underlying mesendoderm cells. We have computed the level of ERK activation in the b6.8, b6.6, b6.7 and b6.5 cells taking into account that b-line cells are in contact both with A-line mesendoderm cells that express a uniform level of FGF and with B-line mesendoderm cells (B6.1, B6.2, B6.3 and B6.4) that express different levels of FGF (Williaume et al. 2021). Surface contacts were measured and FGF concentrations have been estimated from experimental data, as described in the new '*Model of ERK activation in b-line cells'* section (p25-26 of the revised manuscript). As shown in Fig. S2, the model nicely

recapitulates experimentally observed levels of ERK activation in the four cells, which are in line with the fact that only b6.5 expresses *Otx*. These results are described in the new version of the manuscript (lines 200 and following) and further discussed at lines 491 and following.

3. The terminology is complex and is sometimes inconsistent within the paper. If I were writing this paper, I would refer to FGF as an inducer, Ephrin as an inhibitor, Ets as an activator, and Erf as a repressor. The authors do not have to copy my terms, but can they at least look over the terms they are using, particularly with respect to Erf, where I was most confused.
We have changed the terminology according to the Referee's suggestion.

4. Line 35 – Typo: Development biology
Corrected

5. Line 45 – Typo: Allow us to quantify.
Corrected

6. Line 81-83 – Ascidians are described as both “sister group” and “cousins” to vertebrates. It would probably sound better to not use two different family terms to describe the phylogeny.
We have removed the term ‘cousins’ and, in line 82-83, simply state: ‘Although the ascidian tadpole larvae exhibit the basic chordate body plan, these embryos are much simpler than vertebrates.’

7. Line 84 – Non-ascidian tunicates are also chordates with these properties.
Corrected.

8. Line 117 – Punctually sounds strange. It would probably sound better to remove the word.
We have removed the word "punctually".

9. Line 121 – Estimated ligand concentration?
Line 120 in the revised version of the manuscript: In this sentence, we refer to the fact that modelling can be used to analyze the respective effects of changing the number of available FGF receptors or the concentration of the FGF ligand that is perceived by the receptors. They both affect the number of FGF-bound receptors, but in a different way as seen in Eq (5). We have thus kept the initial formulation.

10. Line 126 – “Shape of the ERK-Otx relation” should be rephrased.
Line 124-125 in the revised version of the manuscript: We have removed the word "shape", rephrasing the sentence as " ... to investigate the possible effect of a repressor on the ERK-*Otx* relation."

11. Lines 148-152 – A lot of cell biology here that could use a reference or two.

We have added two references.

Kolch W. 2005. Coordinating ERK/MAPK signalling through scaffolds and inhibitors. Nature Reviews Molecular Cell Biology 6:827–837. DOI: https://doi.org/10.1038/nrm1743, PMID: 16227978

Ornitz DM, Itoh N. The Fibroblast Growth Factor signaling pathway. Wiley Interdiscip Rev Dev Biol. 2015 May-Jun;4(3):215-66. doi: 10.1002/wdev.176. Epub 2015 Mar 13. PMID: 25772309; PMCID: PMC4393358.

These references are mentioned at line 153. We have also added references to Bertrand et al., 2003 and Le Gallic et al., 1999; 2004) at line 157.

12. Line 168 – In the following section?

Line 170 in the revised manuscript. We have changed this into "In the next section".

13. Line 197-203 – What exactly is the basal fluorescence? Autofluorescence from the sample? Non-specific binding of the antibody? Either of these would lead to an artificially high signal. It is interesting that the model falls right in the middle of the experimental data. I think if basal fluorescence was a problem, you would see the model data at the lower end of the experimental values.

In order to minimize the effect caused by autofluorescence, our dpERK immunofluorescence was conducted with Alexa flour 647, for which the 633 nm excitation laser line was used. This laser line hardly induces autofluorescence in ascidian embryos. Therefore, the background fluorescence is most likely due to non-specific binding of antibodies.

In the light of this comment, we feel that "background fluorescence" is more appropriate than "basal fluorescence" and have thus changed throughout the manuscript.

When modelling each set of experiments, we adjusted the value of the background fluorescence (parameter B in Eq (14)) to the experimentally observed average level of fluorescence in cell a6.8, as explained in the presentation of the model, lines 678-680. In the revised manuscript, we have added one sentence at line 209 to clarify this point in the main text.

14. Line 224 – Would it be better to say more linear instead of “less nonlinear”?

Line 238 in the revised manuscript: We have removed "less nonlinear", keeping just the "smoother" that preceded it.

15. Line 232 – Isn’t the ERK activity unchanged in the a6.8 and a6.6 cells (fig 2C)?

Line 246 in the revised manuscript: Corrected.

16. Line 261 – Bertrand et al 2003 should be the reference here. Weaver et al 2022 should go with the Le Gallic papers in the next line.

Line 271 in the revised manuscript: Corrected.

17. Fig 3D – There might not be an elegant solution, but it is hard to distinguish between the different conditions in this graph.
We have changed and increased the size of the symbols to improve the contrast.

18. Line 327 – typo: intuitively logic
Line 347 in the revised manuscript: We have changed "logic" into "expected"

19. Line 432 – Typo: Act should be plural.
Line 467 in the revised manuscript: Corrected.

20. Line 526 – I believe only FGF receptors (not Eph) were shown to be uniform through experimental overexpression. Although it makes the sentence grammatically clumsy, I think this should be stated.
Line 575 in the revised manuscript: This is now clearly stated in the manuscript. We have added a sentence to highlight this point.

21. Line 644 – Repression of an inhibitor is incorrect here. The inhibitor is active.
Line 699 in the revised manuscript: Corrected.

22. Lines 650 – Although both Ets and Erf bind to (and effectively compete for) the same DNA sites, I am not sure if considering Erf as a competitive inhibitor is appropriate. My understanding is that although concentrations are important, at any physiological concentrations, Erf will always repress until it is phosphorylated, in which state it will never repress. Does the model account for this?
Line 704 in the revised manuscript:
Otx expression is modeled by Eq (15); i.e.:

$$\frac{dO}{dt} = v_b + v_o \frac{A_p}{K_a\left(1 + \frac{I}{K_i}\right) + A_p} - kO$$

where *I* stands for non-phosphorylated ERF2. Thus, phosphorylated ERF does not repress *Otx* expression in the model and the presence of non-phosphorylated ERF ($I \neq 0$) always affects the rate of *Otx* expression. At the level of the population of molecules, ERF phosphorylation is not an all-or-none process. According to the model, the level of repression decreases when more ERF molecules get phosphorylated simply because the concentration of non-phosphorylated ERF (I) decreases, which is in agreement with the fact that binding of ERF to the DNA becomes less probable. As seen in Fig. S3B, upon an increase in ERK activation level, there is a rapid and concomitant switch between the phosphorylated status of the activator and the repressor for the values of parameters considered in the model. Thus, in practice, as soon as the activation

level of ERK is large enough, repression becomes very weak and allows for full activation of *Otx* expression by Ets1/2.
We agree however that the use of this expression for an enzymatic competitive inhibition is phenomenological in the case of the regulation of genetic expression. In the future, we plan to develop a stochastic molecular model of Ets1/2 and ERF2 binding/unbinding and phosphorylation.

Reviewer #2:

Major points

1. How exactly has the initial parameter set been chosen? In the text, the authors only state that they used “manual fitting”. It would be informative to explain more clearly the strategy how each of the parameters has been optimized. Specifically, I am left wondering whether the parameters have initially been constrained on the wild type data only, or whether information on perturbed conditions have been used to determine parameters.
The values of the parameters of the two modules of the model were fitted separately. For the first module (model of ERK activation) we first fitted manually the experimental data obtained in ephrin-inhibited embryos (Fig. S1B, S1C), to set the values of the following parameters: V_s, K_1, K_s, K_b, K_{erk}, K_d, [FGF]. We then fitted the experimental data obtained in wild type embryos (Fig. 2A, 2B) to set the values of the remaining parameters: V_{rg}, K_2, K_{rg}, K_e, [ephrin]. To fix the values of the parameters in the model of *Otx* expression (k_{MM3}, K_{MM3}, v_{MM4}, K_{MM4}, k_{MM1}, K_{MM1}, v_{MM2}, K_{MM2}, v_b, v_o, K_a, K_i, k) we manually fitted the experimental data shown in Fig. 3D. Thus, parameters have been constrained using wild type data and NVP-treated embryos only. Experimental data sets obtained from “perturbed” embryos (with dominant-negative forms) were not used to fit the values of the parameters (Figs 2C and 3B) except for parameters A, B, C, and D (please refer to the point 6). Finally, we have added one paragraph explaining the fitting procedure just above Table 1.

2. Related to this point, one of the main messages of the authors is that the bimodal expression of Otx is a consequence of zero-order ultrasensitivity in their model, because “the phosphorylation and dephosphorylation reactions of the two transcription factors are far from saturation” (lines 302 and 303). Is there independent evidence to set the key parameters KMM1-4 as low as they are, or have these values been chose ad hoc? In the latter case, the phenomenon of zero-order ultrasensitivity would qualify more as a prediction from their model, and should clearly be labelled as such.
This point indeed required clarification in the manuscript. The values of $KMM_{1\text{-}4}$ in Eq (16) and (17) have been chosen to fit experimental observations. It appeared that best fit is obtained with values corresponding to zero-order regimes. However, there is no experimental

evidence for zero order ultrasensitivity in the regulation of *Otx* expression and other possibilities, such as cooperative binding of the activator Ets1/2, could be the cause of the ultrasensitive *Otx* response. Indeed, when modifying the evolution equation for Otx to take into account cooperativity in ETS binding (with n_H=2, KMM1=KMM2= KMM3=KMM4=0.3; kMMM1=kMM3=14, see the new section 'Cooperativity in *Otx* activation' on p26-27), the model is able to reproduce the experimental data without zero-order ultrasensitivity. We have added a new figure (Fig. S7) showing this and confirming that model predictions are similar with the two models. This figure is mentioned in the Results and in the Discussion (lines 314, 336, 426, 432, 448, 556, 784). The exact origin of the steep *Otx* versus ERK response is actually the focus of our next project.

3. Fig. 4: The relative changes of Erk activity and Otx expression are hard to decode from the log2-scale that the authors are using. Is it possible to indicate in this plot how big a 20% change of these quantities would be, to make it easier to identify sublinear scaling of the two activities? From the way the data is presented at the moment, I remain to be convinced that the mechanism is robust against changes in parameter values (lines 339 and 428). If I have been missing something here please clarify.
We have followed this suggestion and have added grey backgrounds to indicate 20% and 40% changes. Given that the changes in ERK activation and *Otx* expression levels result from 20% changes in the values of parameters, the model can be considered as robust, except concerning *Otx* expression in the a6.7 cell, as discussed in the text. Most importantly, as indicated in the text, the a6.5>a6.7>a6.6>a6.8 profile of ERK activation and *Otx* expression is maintained in all tested parameter ranges (line 357 and as shown in the new Figure described below).
As another way of assessing the model's robustness, we have performed a new set of simulations where we considered noise on a whole set of values of parameters at the same time. Thus, in the new panel of Fig. 4 (Fig. 4A), parameters related to the FGF (left) or ephrin (right) pathways were randomly changed with the range ± 20% of the default values. Comparison with Fig. S1 shows that the main effect is to increase the variability in both ERK and *Otx* activation levels with little effect on the average values. These figures are now discussed at the beginning of the section "Sensitivity analysis" on p12.

4. The heatmaps in Fig. 5 and Fig. 6 A, B are difficult to understand. Please explain in more detail what the colors mean - my interpretation is that any color value >0 indicates that the first term in the subtraction under the scalebar (nH[Erk(S1)] for Fig. 5A) has a stronger influence in increasing the Hill coefficient than the second one, but please correct me if I'm wrong here. It would be helpful if all color scale bars clearly indicated where the 0-position (no change) is.
The reviewer understood correctly what we were trying to show. Another Reviewer also mentioned that these heatmaps are difficult to understand, so we have tried to clarify their

meaning. The color indicates the difference between the Hill coefficient of the ERK activation response to one parameter (par1) and the Hill coefficient of ERK activation response to another parameter (par2). For example, $n_H[Erk(S_1)]-n_H[Erk(FGF)]$ represents the difference between Hill coefficients of ERK response curve to increasing S_1 (=cell contact surface with FGF-expressing cells) and of ERK response curve to increasing concentration of FGF. And for each column, a third parameter is varied to show the robustness of the observed difference. To clarify these plots, we have introduced three changes. First, we now indicate the 0 position in the scale bar, as suggested by the Reviewer. Second, we have added a new panel (A), which is a graphical explanation of the procedure. This panel is explained in the legend. Finally, we have added a discussion of this new explanatory panel in the text (line 366-371).

Minor points

5. It seems to me that Fig. 1 has been entirely reproduced from Williaume et al., 2021. Even though I acknowledge the necessity of presenting this data for reference, I suggest to make it clearer that the data has been reproduced from a previous paper, and perhaps present it in a less prominent place.
We have modified Fig. 1 in 2 ways: (1) to present Fig. 1B as a scheme and not as real data and, (2) to add a new scheme in Fig. 1C. However, if this looks acceptable for the Reviewers, we prefer to keep it at the same place because it is really important for the understanding of the rest of the manuscript. We acknowledge in the Figure legend that this data is reproduced from Williaume et al, 2021.

6. Parameters A, B, and C, D have been changed in between panels in Figs 2 and 3. What was the reason for this adjustment?
The values of the parameters A, B, and C, D have been changed in between panels in Fig. 2 and 3 because the experimental data shown in the different panels are obtained in different experiments. The background (B) and the maximal (A) levels of fluorescence depend on the experimental setup, which slightly differs from one experiment to the other. Similar considerations hold for C and D in Fig. 3. This is explained in the "Model" section, lines 678-687 and 727-736.

7. Line 241: Word missing: "factor of".
Line 243 in the revised manuscript: Corrected

8. Fig. 3B: Can the authors stagger data from different cells to increase clarity?
We have added a new supplementary figure (Fig. S4), which is referred to in the text at lines 285, 289 and 395.

9. Fig. S2B lacks a Y-axis label.
Fig. S3B in the revised manuscript: Corrected

Reviewer #3:

Major Points:

1. One major weakness of the paper is the lack of novelty in the computational model, as it does not add a novel perspective compared to the mathematical model introduced in earlier works. For instance, extracellular signaling dynamics, both temporal and spatial, are discarded following similar assumptions made in their previously published manuscript. I think the extracellular signaling dynamics, at least in its simplified form, should be considered to differentiate the authors' contributions from previous works. Overall, the paper needs to capture more temporal and spatial (at least for FGF) variation through in-silico modeling, data generation, and analysis.

The experimental data and the different modules that constitute the current model have indeed already been presented in Williaume et al., 2021. However, the modelling modules that were presented in this previous publication were not integrated together, nor calibrated on experimental data, and were used as tools to test concepts and propose hypotheses directly based on experimental observations. As is often the case, it was not possible to present a full analysis of the complete computational model in the first, mostly experimental, publication.

Two key assumptions of the model are (1) that the role played by the spatial distribution of the system can be captured by the surfaces of contact between the cells, and (2) once the embryo reaches the 32-cell stage, FGF and ephrin are homogeneously distributed at cell interfaces.

This last hypothesis is motivated by the observation that all a-line ectoderm cells are in contact with A-line mesendoderm cells that express uniform levels of FGF (Williaume et al., 2021, figure S1A). We have emphasized the biological rationale behind this hypothesis in the manuscript at lines 575-577.

That temporal and spatial variations of FGF can be neglected was also assumed in the model by Guignard et al. (2020) (see around equation (9.15) of their Supplementary Material).

Finally, our sensitivity analyses (Fig. 4) address the impact of variation in the signaling pathways, including the concentration of FGF, on ERK and *Otx* outputs.

2. Downstream activation of signaling pathways and the robustness rely directly on how the extracellular ligand-receptor-regulator dynamics evolve temporarily and spatially. In the process, free-ligand availability is important and often varies spatially over a region because of the heterogeneous extracellular environment. For instance, surface heterogeneity may affect receptor level for the free ligand to bind, or the numerous other signaling regulators may hinder the availability of free ligand to the receptors. These all affect downstream

signaling activation, and a trace of it could be considered in the proposed computational study. For instance, a variation in FGF profile from its currently assumed uniform level requires further exploration to see the changes shape up as the downstream pathways are activated. The four cells of interest are exposed to uniform and constant FGF concentration. But it would be interesting to see the response of the graded FGF exposure to the cells of interest.

As discussed in the previous point, the extracellular level of [FGF] was considered uniform based on experimental observations (Williaume et al., 2021, Fig. S1A) and as in Guignard et al. (2020).

To investigate the point raised by the Reviewer, we performed simulations considering the existence of an extracellular gradient of FGF. We investigated the response of the a-line cells to a graded FGF exposure in a simple configuration that does not correspond to the geometry of the embryo but allows us to explore the response to a graded signal (see figure B below). We considered the anterior a-line cells positioned along a single line and we assume that only a6.5 cell is in direct contact with a mesendoderm cell producing FGF. We considered this mesendoderm cell as the only source of FGF in the system. We also considered that all the a-line cells are equal and therefore have the same area of the cell surface exposed to FGF (S_1). With these assumptions, we computed the FGF gradient needed to reproduce the levels of dpERK immunofluorescence signal measured experimentally for these cells (see figure A below). The experimental data were reproduced by the model when we considered the level of extracellular FGF concentration equal to 20 (a.u.) for a6.5 cell, 7 for a6.7 cell, 3 for a6.6 cell and 1 for a6.8 cell. The extracellular FGF concentrations sensed by the different cells are shown as blue dots in figure B. In this analysis we assumed each cell has a dimension/length of 30 μm.

In general, to have a stable gradient in one dimension, the concentration of the molecule that diffuses (c) must satisfy the following differential equation (Wartlick et al., 2009):

$$\frac{\partial^2 c}{\partial x^2} = \frac{k}{D} c$$

Where D is the diffusion coefficient and k is an effective linear degradation rate. The solution of this differential equation has the form:

$$c(x) = c_0 e^{-\frac{x}{\lambda}} \quad (*)$$

Where $c_0 = \frac{J_0 \lambda}{D}$ is the concentration at x=0 and $\lambda = \sqrt{D/k}$ is the decay length i.e. the length over which the concentration decreases by a factor of e. The solution above is obtained assuming a constant flux of molecules J_0 at the source of the gradient (positioned at x=0) and no flux at the boundary. Eq (*) is an approximation valid when the dimension of the system is much larger than the decay length λ.

We used expression (*) to fit the blue points in Fig. B. From the fit we obtained an initial concentration c_0=56 (a.u.) and a decay length $\lambda = 30\ \mu m$. These values correspond to a degradation rate k ≅ 0.0018 s^{-1} (obtained assuming c_0= 56 (a.u.) and D ≅ $1.6\ \frac{\mu m^2}{s}$ as in Müller et al., 2013). The time needed for the gradient to be established in this simple configuration

is around 25 minutes (computed as in Berezhkovskii et al. 2010), which is almost equal to one cell cycle in the ascidian embryo.

From this analysis we conclude that a morphogen gradient system is compatible with the parameter set of our model to recapitulate the ERK activation profile observed in the a-line ectoderm cells. However, this relies on an embryonic configuration that does not correspond to ascidian embryos, where all the a-line cells are in contact with FGF-expressing mesendoderm cells. Thus, the most prominent gradient of FGF would be actually perpendicular to the one assumed in this theoretical configuration. There might be a small FGF gradient within the field of ectoderm cells due to the spherical configuration of the embryo, but it would not be steep enough to account for the a6.5>a6.7>a6.6>a6.8 profile of ERK activation. Although we concede that a very slight FGF gradient could contribute in some way to the pattern of ERK activation observed, we have no evidence for this and it is not necessary to explain our data.

In contrast, as shown in our study, the measured differential area of cell surface exposure to FGF ligands in the ascidian neural model are sufficient to recapitulate experimental observations.

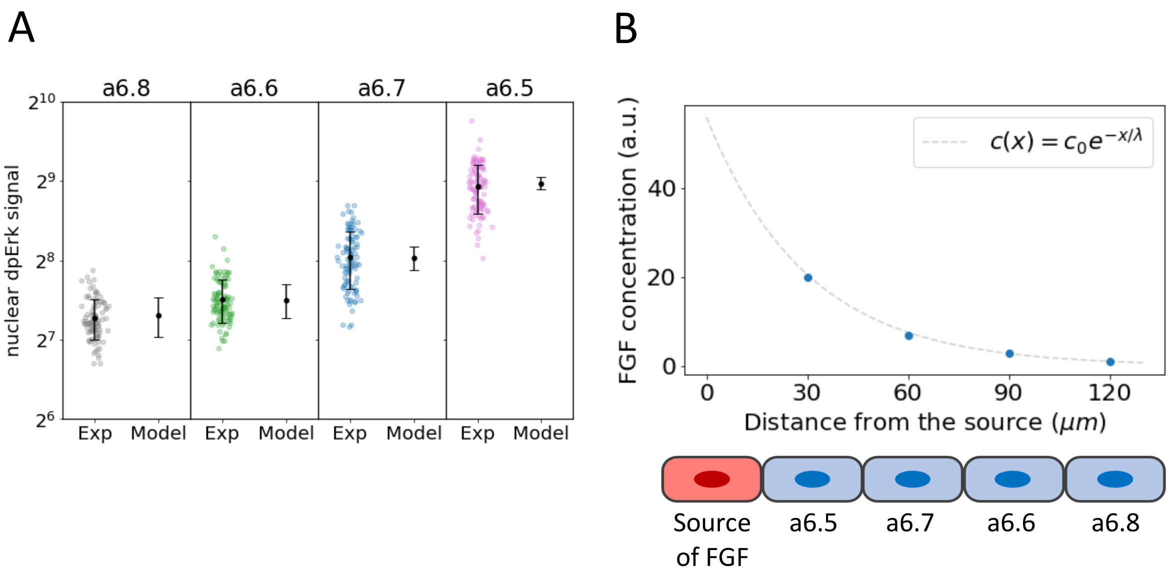

References:

- Berezhkovskii AM, Sample C, Shvartsman SY. How long does it take to establish a morphogen gradient? Biophys J. 2010 Oct 20;99(8):L59-61. doi: 10.1016/j.bpj.2010.07.045. Erratum in: Biophys J. 2010 Nov 17;99(10):3505. PMID: 20959075; PMCID: PMC2955507.

- Müller P, Rogers KW, Yu SR, Brand M, Schier AF. Morphogen transport. Development. 2013 Apr;140(8):1621-38. doi: 10.1242/dev.083519. PMID: 23533171; PMCID: PMC3621481.

3. How about varying embryo volume or cell volume, or both, to see if the EGF>ERK>ETS>Otx expression remains invariant? Authors may consider using relative-volume to normalize any volumetric variation at the cellular or embryo level and demonstrate the relationship between the contact areas and the underlying neural induction.

For the calculations of cell surface contact, relative area was used. Small variations in cell volume would result in slightly different values of the reaction rates/parameters of the model. Thus, variations in cell volumes have been indirectly investigated in the sensitivity analysis of Fig. 4. Indeed, to consider the point of the Referee more specifically, we have performed new simulations to compute the levels of ERK activation in the different cell types obtained by randomly choosing the values of the parameters linked to the FGF pathway ([FGF], K_d, K_1, K_s, V_s, R_T, on the left) or the ephrin pathway ([ephrin], K_e, K_2, K_{rg}, V_{rg}, Q_T, in the middle) in the range ± 20% of standard values. These results are shown in the new Fig. 4A and discussed in the text (lines 328-336). The variability in the level of ERK activation obtained by varying the FGF pathway related parameters is much higher than that obtained by varying the ephrin pathway related parameters, suggesting that the former parameters have a larger influence on the level of ERK activation than the latter parameters. In both cases, there is a larger variability in ERK activation levels than those obtained with the optimal parameter values (compare with Fig. S1A), however, it is important to note that the ERK activation and *Otx* expression profiles are maintained, indicating that the process is robust against small changes in cell volumes.

4. [Line 243 to 244, Line 285 to 285] How a moderately nonlinear relationship between surface contacts with FGF-expressing cells and ERK activation transforms into a highly nonlinear relation between Otx expression and ERK activity may depend on numerous other factors that are not considered in the analysis. For instance, the intracellular dynamics are assumed in this study to be linearly cascading down to the next level, which is just an assumption considered previously and not explored further in this mechanistic study. Authors could expand the analysis to accommodate the intracellular dynamics to channel down the nonlinearity part to the interplay between ERF, ETS, Ephrin, and GATA interactions. Interestingly, GATA is known to promote ERK signaling, thereby a potent candidate for becoming the source of additional nonlinearity.

Thank you for pointing this out. Indeed, there are experimental evidence indicating an ERK-dependent regulation of GATA activity during *Otx* transcriptional activation (Bertrand et al, 2003) and we agree that this is interesting to analyze further. We are currently collecting experimental evidence for the role of Gata.a during early neural induction in ascidians. However, we believe that this experimental evidence, which will take time to acquire, is beyond the scope of the current manuscript.

The mechanism that translates ERK activity to *Otx* expression is described by nonlinear enzymatic reaction laws. To obtain the highly nonlinear dependence in the *Otx versus* ERK relation (Fig. 3D), we considered that K_1 and $K_2 << 1$ to induce zero order ultrasensitivity. At this stage, as discussed at lines 549-561, this is a theoretical hypothesis. Since there is no

experimental evidence for zero order ultrasensitivity in the regulation of *Otx* expression, other possibilities, such as cooperative binding of the activator Ets1/2, could be the cause of the ultrasensitive *Otx* response. To investigate the latter possibility, we have modified the evolution equation for *Otx* to take into account cooperativity in Ets1/2 binding (see section 'Cooperativity in *Otx* activation' on p2). The modified model is also able to reproduce the experimental data (see Fig. S7). We have added sentences to describe these results at lines 309-314, 425-434.

5. [Line 178 to 185] The modeling assumptions and other descriptions require further clarification. For instance, exact simulation details to achieve steady-state solutions of Eq. 1-13 that include both Ordinary Differential Equations (ODE) as well as algebraic expressions for the downstream activation are missing. How long the dynamics were allowed to evolve to reach a steady state? Also, authors should clarify the units of each quantity being computed or measured. If the analysis considers dimensionless form, they should explicitly mention that (See line 186 to 187 of the manuscript, computed Erk* is dimensionless).

As described in the Modeling section at lines 745-748, all equations were solved analytically at steady state (i.e. all time derivatives equal 0). All the differential equations and algebraic expression used in the simulations are described in the Modeling section. Codes and experimental data are available on Github.

In the absence of experimental data about the values of the parameters, we consider that all parameters have arbitrary units (or are dimensionless). This is mentioned in the legend of Table 1.

6. [Line 194 to 196] The model used an extracellular FGF level of KD = 25/5, which produced a maximum ERK activation level of 0.18 in an a6.5 cell. Any rationale for the specific KD/5? Is it to avoid receptor saturation? At what level do we encounter receptor saturation? Does it have anything to do with the areas of cell-contacts we have? Also, the dependency of FGF level and the maximal ERK activation could be explored in addition to an artificial overexpression of FGF simulation.

I think the authors could have exploited the flexibility of an in silico analysis to provide a more detailed picture of extracellular and intracellular coupling of FGF-mediated ERK activation and the subsequent activation of the downstream.

The value of K_D was chosen to fit the experimental observations. With the standard values we used (see Table 1), receptors are far from saturation: only 1/6 of the receptors are bound to the ligand. Receptor saturation can be obtained with Kd << [FGF]. This is visible in the figure here below:

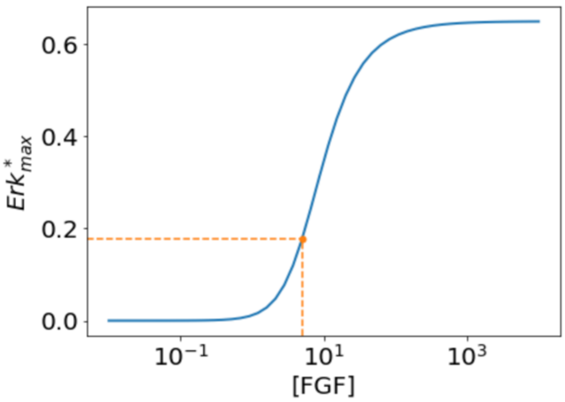

ERK activation levels depend on cell surface contact and on [FGF] in a very different way (see model equations or Figure 4B in the sensitivity analysis). We could not find a good agreement between the observations and the model when assuming receptor saturation, even when considering other receptor densities or cell surface contacts.
Actually, the non-saturated status of FGF receptors is in line with experimental observations. dpERK IF signal levels detected in a6.5 cells can increase by a factor of ~2 in a6.5 cells of ephrin-inhibited embryos, while by a factor of ~5 in maximally responding cells of ectodermal explants treated with high concentrations of exogenous FGF (Williaume et al., 2021).
This point was discussed at the beginning of page 17. We have extended this discussion at lines 480-483.

7. [Line 230 to Line 232] Authors report a reduction in ERK activity in cells for a reduction in FGF receptors. Is this reduction occurs because of receptor saturation? If yes, authors may want to see the dose-dependent response of the receptor occupancy, a mechanistic model of the FGF, FGFR, and the subsequent activation of the downstream would provide a better understanding of the underlying reasons.
As discussed in the previous point, FGF receptors are not saturated in our model.
The reduction of ERK activation levels in a6.5 and a6.7 cells (and to a lesser extent in a6.6 cells) observed in Fig. 2C (dnFGFR) does not occur due to receptor saturation but due to a reduction of the total number of FGF receptors present on the cell surface (see Eq (5)). In the model, the [FGF] imposes the fraction of receptors that are bound to FGF, while the number of receptors scales the maximal response (see Eq. 5 and 6). In normal conditions we considered 2000 FGF receptors per cell. To model the effect of the injection of a dominant negative form of the FGF receptor, we reduced the number of total FGF receptors to 100.
To verify that our results do not depend on the simplified model of the ERK pathway that we use, we have performed simulations based the Ferrell's model that provides a detailed description of the ERK pathway (Huang and Ferrell, 1996). The model describes a generic mitogen activated protein kinase (MAPK) pathway and consists in 16 odes and 7 algebraic equations. In our case Raf is the MAPKKK, MEK the MAPKK and ERK the MAPK. We used our model to compute the fraction of Ras-GTP present in each cell (Eq (13)) and we subsequently used the model developed by Huang and Ferrell to obtain the fraction of doubly

phosphorylated ERK. We computed the level of dpERK IF signals with Eq (14). Here below, we schematize this detailed model and compare the results obtained with this approach with the levels of ERK fluorescence measured experimentally. The model reproduces experimental data, showing that the ERK pathway can be safely described/replaced by a Hill function, as we did in our manuscript. The values of the parameters of the Ferrell's model used to reproduce the experimental data are indicated in the Table here below. All the values of the parameters fall in the range explored by Huang and Ferrell in their paper, except for the total concentration of the enzyme E_2 (E_{2tot} = 0.003 μM, which is slightly higher than the maximum value considered by Huang and Ferrell: 0.0015 μM). E_{1tot} was taken to represent the level of input stimulus to the cascade and was computed as T*Ras-GTP_{tot} where T was obtained solving Eq (13) and Ras-GTP_{tot} was assumed to be 0.001 μM.
At lines 498-501 and 688-689, we have thus mentioned that theoretical predictions remain unchanged when using Ferrell's model.

Enzyme	Assumed concentration (μM)
Raf_{tot}	0.01
E_{1tot} (Ras-GTP)	Variable
E_{2tot}	0.003
MEK_{tot}	0.25
MEK P'ase_{tot}	0.0015
ERK_{tot}	0.25
ERK P'ase $_{tot}$	0.025
Rate constant	Assumed value
a_i (i ranging from 1 to 10)	1000 (s^{-1}*μM^{-1})
k_i (i ranging from 1 to 10)	800 (s^{-1})
d_i (i ranging from 1 to 10)	700 (s^{-1})

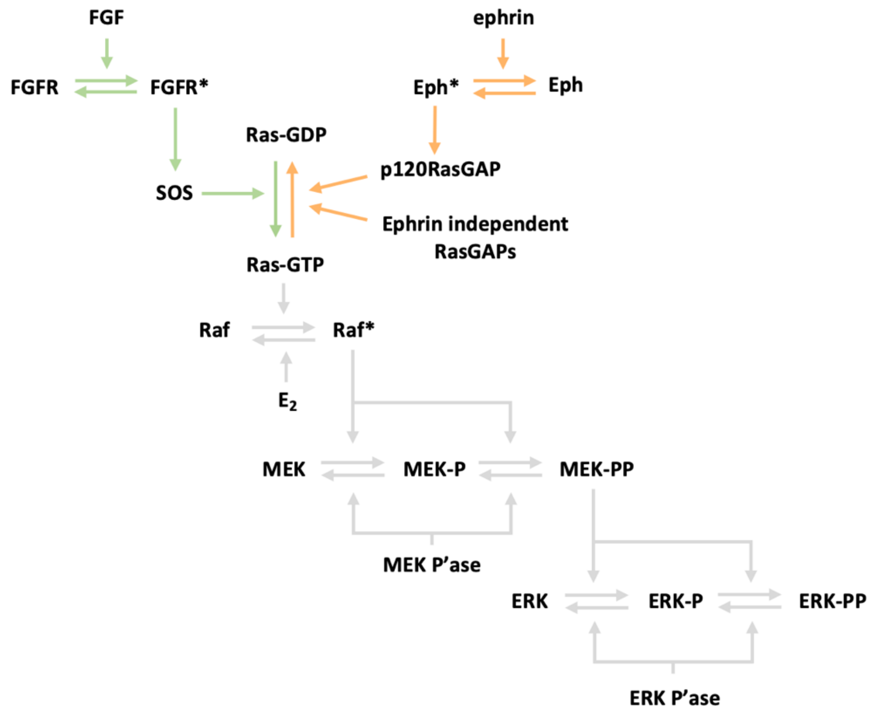

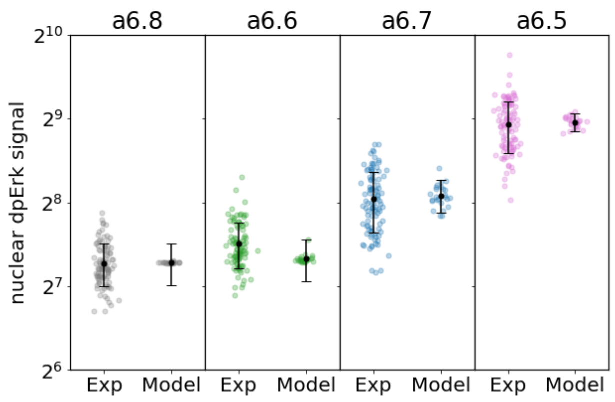

8. [Line 323 to Line 324] Authors reported a stronger control by FGF-receptor pathway on the Otx activation than the control exerted by the ephrin/Eph. Can we have a ratiometric presentation of this stronger for parameters affecting the strength of the control? Is the ephrin/Eph is dispensible at any condition? For instance, overexpression of EGF or through the role of GATA that strengthens the ERK activation further? What happens to this stronger control if concentrations of EGF and/or ephrin changes in combination?

The stronger effect of the FGF receptor pathway on ERK activation/*Otx* activation is shown in Fig. 4B-C. More evidence of this can be seen in the new Fig. 4A that shows the predicted levels of ERK activation in the different cell types obtained by randomly varying the values of the parameters linked to the FGF pathway ([FGF], K_d, K_1, K_s, V_s, R_T, on the left) or the ephrin pathway ([ephrin], K_e, K_2, K_{rg}, V_{rg}, $Q_{T,}$ in the middle) in the range ± 20% of standard value. The

variability in the level of ERK activation obtained in the first case is larger than that obtained in the second one.

As shown in Fig. S1B, S1C (NVP-treated embryos) and discussed in the text at lines 232-238, the presence of ephrin is not necessary to generate the a6.5>a6.7>a6.6>a6.8 profile of Erk* activation in the four cell types but is important to prevent ectopic expression of *Otx* in a6.7 cells (see Fig. 3B-D and text lines 285-292).

If we overexpress FGF ([FGF]=500 instead of 5 in the model), in the presence or in the absence of ephrin, all cell types express *Otx.*

9. [Line 349 to Line 352] Sensitivity analysis on surface contact of ephrin-expressing cells and ephrin-concentration requires further clarification. How a difference between hill-coefficient manifests the higher sensitivity for surface contact is not clear from the manuscript. Perhaps, the authors consider the level of cooperativity exerted by each of these competing factors. If so, a better explanation would help readers to relate the hill-coefficient difference to the degree of sensitivity. Also, modeling details of the sensitivity analysis are insufficient for readers to follow and reproduce the study.

All the modelling details required to reproduce the analysis are described in the Modeling section. By higher sensitivity we mean that, in the steepest part of the S-shape response curve, cells react relatively more (i.e are more sensitive) to small changes in input stimulus. This is reflected by a higher Hill coefficient. Thus, higher Hill coefficients indicate a bigger change in output for a given change in input in the steepest part of the response curve. So ERK activation levels are more sensitive to S_1 than to FGF ligand concentration and this is reflected in the Hill coefficient of ERK-to-S_1 being higher than the Hill coefficient ERK-to-FGF. We have tried to further clarify the procedure of the Hill coefficient analysis presented in Figure 5 with a schematic drawing panel in Fig. 5A (with explanation in legend).

For the rest of the figure, the color "heat" indicates the difference between the Hill coefficient of the ERK activation response to one parameter and the Hill coefficient of ERK activation response to another parameter. For example, n_H[Erk(S_1)]-n_H[Erk(FGF)] represents the difference between Hill coefficients of ERK response curve to increasing S_1 (=cell contact surface with FGF-expressing cells) and of ERK response curve to increasing concentration of FGF. And for each column, a third parameter is varied to show the robustness of the observed difference. To clarify these plots, we have introduced three changes. First, we now indicate the 0 position in the scale bar, as suggested by the Reviewer 2. Second, we have added a new panel (A), which is a graphical explanation of the procedure, as mentioned above. Finally, we have added a discussion of this new explanatory panel in the text (line 366-371). We hope that this is now clear for the reviewer.

10. Authors could consider a rigorous kinetic screen (over a physiologically relevant parameter range) in the intracellular regulation of Otx expression through the two competing pathways– ERF2 and ETS.

The kinetic dependence of the transformation of moderately non linear extracellular coupling of FGF and ERK to a strong nonlinear dependence of Otx on the ERK activation would be interesting to see.
We apologize but we do not understand what the Reviewer means by “a kinetic screen”.
The analysis of the dependence of *Otx* expression on ERK for different values of the parameters is performed in Fig.3, 4, 6, S3, S6 and S7.
A systematic analysis of the effect of changing the values of the parameters on ERK activity and *Otx* expression is done for the sensitivity analysis (Fig. 4). Finally, as described in our response to Point 4, we have added a new model considering cooperativity in Ets1/2 binding, which faithfully recapitulates the experimental observation in first-order regime (S7 Fig).

11. [Line 520 to 522] Authors claimed to combine the previously developed three modules and calibrated with previously obtained experimental data. This does not portray any clear contribution in the mechanistic model building, as the model remains incapacitate to portray the signaling dynamics.
The Reviewer refers to the sentence: “Here we combine these modules and calibrate the values of the parameters to compare the outcome of the full model with experimental results.”
The adequacy of our modelling of the signaling dynamics is discussed in the points 1, 2, 6 and 7 here above.

12. [Line 527 to 528] Were the FGF and Eph receptor density similar? Did the authors consider alternative combinations of receptor density to capture the impact on the interplay between the FGF and ephrin signaling? It is important.
Receptor density enters in the model via parameters R_T and Q_T that represent the total numbers of FGF and ephrin receptors, respectively. R_T and Q_T enter into the model via Eq (6) and Eq (10), where they are multiplied by S_1 and S_2, respectively. Thus, changes in R_T and Q_T are equivalent to changes in S_1 and S_2, and all have been investigated in the sensitivity analysis (Fig. 4). We have added one sentence in the legend to Fig. 4 to emphasize this point.

13. Table 1 data does not show units of any quantity.
We now mention in the legend to Table 1 that all units are arbitrary (p27-28). We thank the Reviewer for reminding us to do so.

14. The authors claim that the data obtained through the best agreement with experimental observations, most (if not all) of which are from the previously published paper. The sensitive parameters could be considered in range to perform a kinetic screen (as suggested above) for an exhaustive in-silico response of the system. Precisely, the KD for phosphorylation of ETS turns out to be about 1200 (KD = K_on/K_off) as per the reported value in the paper, which indicates a very low binding affinity. A screen over a wide KD range may explain the role of Phosphorylation process (details of which are discarded in the mathematical model) better in the final ON/OFF induction.

The K_D for the phosphorylation of ETS (named K_{MM3} in the model, see Eq (16)) is 0.05 (as reported in Table 1). This value, which is much smaller than 1, agrees with our hypothesis of zero order ultrasensitivity (see point 4 here above). The impact of changing the value of K_{MM3} is investigated in Fig. 4 and in Fig. S6. For further modelling of *Otx*, please see Fig S7.

15. Also, receptor count requires cellular volume, which is not clarified anywhere in the manuscript.
In the absence of experimental data for ascidians, we used R_T = 2000 in line with values reported for mammalian cells (see Filion and Popel, 2004). As this represents the number of receptors on the surface of the cell, its value can be defined without resorting to cellular volume. We have added one sentence to clarify this point at lines 627-628.

16. A few additional short points:

- Cell volume used to count R_{T} = 2000 to be clarified.
  Please see our response to Point 15.
- Knockdown of receptor level considers 100 receptors for FGF and 10 for Ephrin are different without any explanation. Why they are different? What were the normal receptor density considered for FGF and Ephrin? [Line 272 to 276]
  They are different to better fit the experimental data. From an experimental point of view, there is no reason to assume that the effective reduction in the number of FGF receptors in the dnFGFR-injected embryos should be the same as the effective reduction in the number of ephrin receptors in the Eph3ΔC-injected embryos.
  The standard number of receptors (R_T, Q_T) in each cell was 2000 (as indicated in Table 1).
- Referring to equation in the manuscript does not follow any uniform style– sometimes it is Eq (X), Eq. X. Authors should choose a uniform style.
  This has been corrected.
- In line 647, a bracket is missing for the maximal Otx expression rate equation.
  This has been corrected.
- Authors could arrange the mathematical equations considering their relevance (extracellular, intracellular) following their appearance in the top-down hierarchy of the singaling pathways.
  Although we understand the Reviewer's point of view, we feel that the current presentation of the equations allows us to follow a more logical presentation. However, this could of course be modified if required.
- Figure 5 is difficult to follow– authors should clarify further the way the hill-coefficient difference for alternative parameters are summing up to higher degree of sensitivity.
  We agree with this remark. As discussed in Point 9, we have significantly improved the clarity of the presentation of Fig. 5.
