# Supplementary material for: Model of neural induction in the ascidian embryo
Source: PLoS Comput Biol. 2023 Feb 3;19(2):e1010335. doi: 10.1371/journal.pcbi.1010335 (PMC9931142; doi:10.1371/journal.pcbi.1010335)
Supplement: Attachment — Submitted filename: review_on_PCOMPBIOL-D-22-00981_R1.pdf [file pcbi.1010335.s011.pdf]

I want to thank the authors for their vivid responses to the queries and concerns raised in version 1 of the same manuscript. After reviewing the authors' detailed review replies, I consider that they have provided substantial explanations (and figures) to clarify doubts and concerns raised earlier. For instance, a size variation on the embryo or cell-volume level explanations, as provided in the revised manuscript, is pertinent. The relevant figure looks okay to me. Also, it is good that the authors have clarified Figure 5 and the relevant discussion part, thereby improving the readability and clarity of the story authors intend to portray here. I also have found the authors to be rigorous in dealing with a few queries made during the review—for instance, the FGF gradient as explained using the simple diffusion-decay mechanism. The authors mention that the time course of diffusion may be insufficient for the ascidian embryo cycle. However, modulator activity may upregulate the diffusivity of a species during gradient formation. While I take this argument of insufficient time-course of gradient formation argument as "subject to the further investigation", overall, the response from the authors to this issue is appreciated. Authors can consider adding the simplistic view of the FGF gradient formation and the relevant argument in the supplemental information. A few minor suggestions:

Minor suggestions:

[Modeling Equations] Authors could consider listing out the mathematical equations following the extracellular to intracellular top-down hierarchy to allow readers to relate the complete signaling cascade from the FGF to the *Otx* expression.

[Line 180 to 182] Exact dependency expression (mathematical) between S1 and S2 would help the readers.

[Line 186-187] any reference or supplemental figure explaining the linearity between the Erk* and the fluorescence data would be useful.

[Line 210-211] dpERK-IF or dpERK IF; author should follow a uniform style.

[Line 219 to 220] “Inverse relationship between”—the term Inverse might be confusing. Authors can consider “opposite role” or other phrases.

[Line 225 to 229] Author should specifically mention the Figure number readers need to follow from Williaume et al., 2021 paper.

[Line 252] Period is missing at the end of the line.

[Line 258 to 259] The “moderately nonlinear” trend should relate with a parametric value here to provide a sense of “strongly nonlinear” vs. “moderately nonlinear” dependency.

[Line 280 to 283] Authors could consider rephrasing this portion as the meaning gets convoluted here.

[Line 293] The bracket ‘(‘ part does not end.

[Line 484 to 485] dpERK-IF or dpERK IF; author should follow a uniform style.

[Line 648 to 649] The term Ke is confusing. What is its unit? Authors should clarify further.

[Line 722 to 724] Ap and Ip, as in line 722, should appear as Ap and Ip in line 724 for a better readability.
